# Detection of infective endocarditis with [^64^Cu]Cu-DOTATATE positron emission tomography/computed tomography: a case series

**DOI:** 10.1093/ehjcr/ytae431

**Published:** 2024-08-20

**Authors:** Katra Hadji-Turdeghal, Marie Øbro Fosbøl, Philip Hasbak, Andreas Kjaer, Lars Køber, Rasmus Sejersten Ripa, Emil Loldrup Fosbøl

**Affiliations:** Department of Cardiology, Copenhagen University Hospital - Rigshospitalet, 2100 Copenhagen, Denmark; Department of Clinical Physiology and Nuclear Medicine, Copenhagen University Hospital - Rigshospitalet, 2100 Copenhagen, Denmark; Cluster for Molecular Imaging, Department of Biomedical Sciences, University of Copenhagen, 2200 Copenhagen, Denmark; Department of Clinical Physiology and Nuclear Medicine, Copenhagen University Hospital - Rigshospitalet, 2100 Copenhagen, Denmark; Department of Clinical Physiology and Nuclear Medicine, Copenhagen University Hospital - Rigshospitalet, 2100 Copenhagen, Denmark; Cluster for Molecular Imaging, Department of Biomedical Sciences, University of Copenhagen, 2200 Copenhagen, Denmark; Department of Cardiology, Copenhagen University Hospital - Rigshospitalet, 2100 Copenhagen, Denmark; Department of Clinical Medicine, University of Copenhagen, 2200 Copenhagen, Denmark; Department of Clinical Physiology and Nuclear Medicine, Copenhagen University Hospital - Rigshospitalet, 2100 Copenhagen, Denmark; Cluster for Molecular Imaging, Department of Biomedical Sciences, University of Copenhagen, 2200 Copenhagen, Denmark; Department of Clinical Medicine, University of Copenhagen, 2200 Copenhagen, Denmark; Department of Cardiology, Copenhagen University Hospital - Rigshospitalet, 2100 Copenhagen, Denmark; Department of Clinical Medicine, University of Copenhagen, 2200 Copenhagen, Denmark

**Keywords:** Infective endocarditis, Prosthetic valve endocarditis, Native valve endocarditis, Cardiac imaging, Nuclear imaging, [^64^Cu]Cu-DOTATATE, PET/CT, Case reports

## Abstract

**Background:**

Infective endocarditis (IE) is a serious and fatal condition, with prosthetic valve endocarditis representing the worst prognosis. The recommended nuclear imaging modality 2-deoxy-2-[^18^F]fluoro-D-glucose positron emission tomography/computed tomography ([^18^F]FDG PET/CT) has limitations. In this case series, we present two patients with IE scanned with a novel PET tracer [^64^Cu]Cu-DOTATATE ([^64^Cu]Cu-[1,4,7,10-tetraazacyclododecane-*N*,*N*′,*N*″,*N*‴-tetra acetic acid]-d-Phe1, Tyr3-octreotate).

**Case summary:**

An 84-year-old female patient (Patient 1) with a biological mitral valve prosthesis (MVP) was admitted acutely from the outpatient clinic. Transoesophageal echocardiography showed vegetations on the MVP. The patient underwent [^64^Cu]Cu-DOTATATE PET/CT, which showed uptake at the site of infection. The patient underwent surgical valve replacement. The post-operative period was without significant complications, and the patient was discharged home. In another case, a 72-year-old male patient (Patient 2) with a medical history of mild mitral valve stenosis, aortic valve stenosis, and gastrointestinal stromal tumour was admitted to the hospital for back and abdominal pain and subfebrile episodes. Transoesophageal echocardiography showed large vegetations in the native aortic valve. The patient underwent [^64^Cu]Cu-DOTATATE PET/CT, which showed no uptake at the site of the suspected infection. The patient underwent surgical valve replacement. The post-operative period was characterized by *Candida albicans* sternitis, and after prolonged hospitalization, the patient died of respiratory failure as a complication of sepsis.

**Discussion:**

In conclusion, this is the first case series presenting two patients with definite IE (modified Duke criteria), who were scanned with the novel [^64^Cu]Cu-DOTATATE PET/CT. Patient 1, with endocarditis in the MVP, showed an uptake of the tracer, while Patient 2, with native aortic valve endocarditis, did not show any uptake.

**Learning points**

- [^64^Cu]Cu-[1,4,7,10-tetraazacyclododecane-*N*,*N*′,*N*″,*N*‴-tetra acetic acid]-d-Phe1, Tyr3-octreotate positron emission tomography/computed tomography ([^64^Cu]Cu-DOTATATE PET/CT) is a novel radioactive tracer with potential advantages in the detection of infective endocarditis. The tracer has low cardiac uptake and does not require specific patient preparations.
- In this case series, two patients with IE were examined, and [^64^Cu]Cu-DOTATATE PET/CT successfully showed positive uptake in prosthetic valve endocarditis, but not in native valve endocarditis.

## Introduction

Infective endocarditis (IE) is a potentially fatal condition,^1^ with prosthetic valve endocarditis (PVE) representing the worst prognosis. Prosthetic valve endocarditis accounts for an increasing proportion of the overall IE cases.^2,3^ The diagnosis of PVE is sometimes challenging, and transoesophageal echocardiography (TEE) can be inadequate due to, for example, shielding, shadowing, or hyper-reflectance from the prosthesis—especially in newly operated patients.^4^

Positron emission tomography/computed tomography with 2-deoxy-2-[^18^F]fluoro-D-glucose ([^18^F]FDG PET/CT) is recommended by the European Society of Cardiology in patients with PVE, when TEE is inconclusive,^5^ but has limitations regarding sensitivity and specificity.^6^ Patient preparation before [^18^F]FDG PET/CT is meticulous, requiring strict dietary restrictions and 12 h fasting,^7^ which is difficult for some hospitalized patients to follow, especially those with diabetes. Even with optimal preparations, up to 30% of the patients will have an inconclusive examination due to inadequate suppression of physiological [^18^F]FDG uptake in the normal myocytes. Further, [^18^F]FDG PET/CT has no or only a limited role in diagnosing native valve endocarditis (NVE), and in this context, we may also benefit from better additional diagnostic tools.^5^

[^64^Cu]Cu-[1,4,7,10-tetraazacyclododecane-*N*,*N*′,*N*″,*N*‴-tetra acetic acid]-d-Phe1, Tyr3-octreotate ([^64^Cu]Cu-DOTATATE) PET/CT is a radiolabelled somatostatin analogue with a high binding affinity for somatostatin receptor subtype 2 (SSTR_2_).^8^ The tracer is currently used to diagnose and monitor patients with neuroendocrine neoplasms, which express SSTR_2_ receptors.^9^ The SSTR_2_ receptors are also abundantly expressed on the surface of pro-inflammatory macrophages.^10^ In the last decade, [^64^Cu]Cu-DOTATATE has emerged as a promising tracer for early detection of atherosclerotic plaques.^11,12^ Macrophages are also part of the inflammatory response in relation to IE and are also present in the heart valves.^13^ A distinct advantage of [^64^Cu]Cu-DOTATATE PET/CT over [^18^F]FDG PET/CT is its low physiological myocardial uptake; further, [^64^Cu]Cu-DOTATATE PET/CT does not require special patient preparations, such as fasting or dietary restrictions. The [^64^Cu]Cu-DOTATATE PET/CT could be a potential alternative to [^18^F]FDG PET/CT.

Two patients with IE were examined with [^64^Cu]Cu-DOTATATE PET/CT: one with PVE and one with NVE to explore whether [^64^Cu]Cu-DOTATATE PET/CT shows uptake in infected vegetations.

## Summary figure

**Figure:**
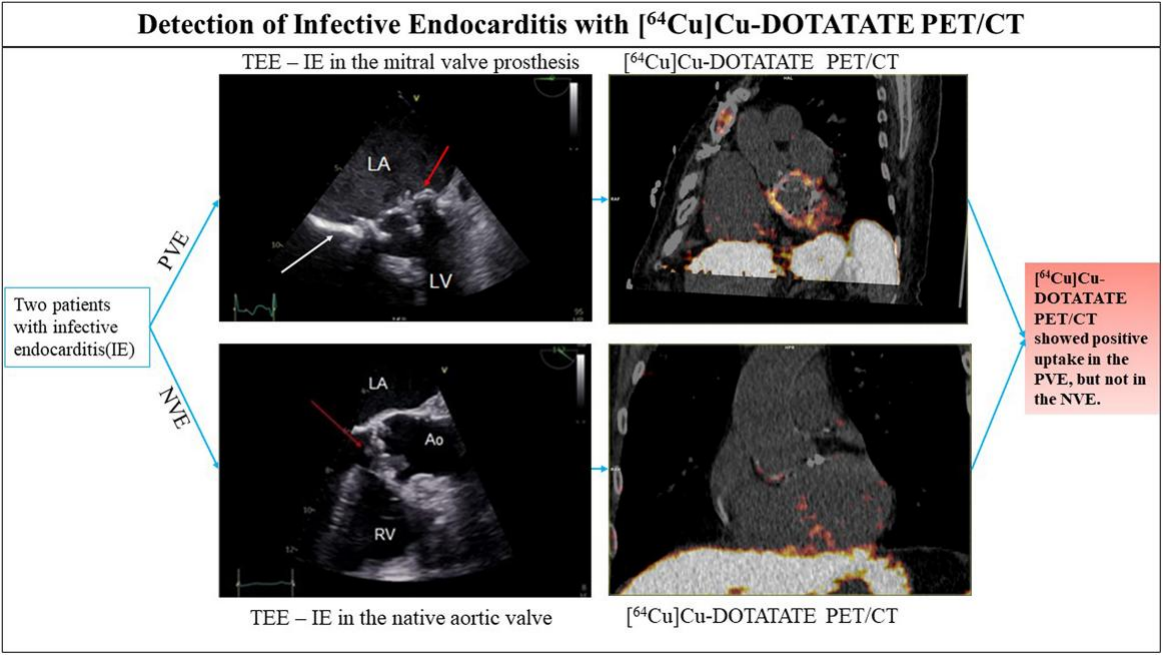


## Case presentation

### Timeline: Patient 1

An 84-year-old female patient with a medical history of a biological mitral valve prosthesis (MVP; surgery in 2008 due to rheumatic mitral regurgitation) and atrial fibrillation (Apixaban 2.5 mg twice a day) was referred to an outpatient clinic by her general practitioner (GP)—as an urgent case and was seen within a week—due to dyspnoea, weight loss of 10 kg, reduction of her functional status, and fatigue over several months. Prior to this, the GP had referred the patient for a CT of the thorax and abdomen, but there were no signs of malignancy or thrombosis (examined 8 days before the referral). The most recent transthoracic echocardiography (TTE; 9 months before admission) showed a left ventricular ejection fraction (LVEF) of 40–45% and MVP *in situ* with moderate stenosis. The patient was married and self-reliant in daily activities with a high physical and cognitive functional status. From the outpatient clinic, the patient was admitted to the cardiology ward. The physical examination revealed a diastolic murmur. A detailed timeline of the patient’s progress during hospitalization is given in *Table 1*.

**Table 1:** Timeline Patient 1

Time	Event
Initial examination	The patient was referred to the outpatient clinic by her GP due to dyspnoea, fatigue, and weight loss (10 kg). The examination revealed a systolic murmur. TTE showed severe stenosis in the biological mitral valve and a hyperdynamic LVEF. The patient was referred to urgent TEE in the outpatient clinic
Day 1	TEE showed a hyperdynamic left ventricle, mitral valve severely stenotic. The examinator suspected thrombosis or an IE of the MVP. The patient was admitted to the cardiology ward for further examination
Day 3	Blood cultures: 12 bottles out of 12 with *Streptococcus salivarius* subspecies
Day 4	Transferred to the tertiary centre for surgical assessment. [^64^Cu]Cu-DOTATATE PET/CT performed. Endocarditis team conference—Euro Score 2 of 9.8—referred for surgical valve replacement
Day 8	Surgery—a new biological prosthesis was inserted (Epic 31). The infected prosthesis showed no bacterial growth
Day 8	Transferred to the ICU after the operation. Eight episodes of seizures. Loaded with Keppra 60 mg/kg (3 g) and diazepam 10 mg. Referred for an MRI of the brain
Day 10	Neurological progress—started physiotherapy
Day 11	Transferred to the cardiology ward—treated with i.v. meropenem and rifampicin
Day 16	MRI (brain): possible embolus in the right frontal lobe—seen by the neurologist and interpreted as an older embolus. Seizures were interpreted as related to the surgery—and follow-up with EEG at the neurological outpatient clinic was planned
Day 17	Rifampicin changed to moxifloxacin due to nausea. Transferred to the local hospital
Day 29	Dental examination—no oral infectious foci
Day 31	[^18^F]FDG PET/CT—no infectious foci. Uptake in colon—referred for colonoscopy
Day 33	New TEE—normal LVEF, MVP *in situ*. Changed from i.v. antibiotics to peroral treatment: tablet amoxicillin 1 g × 4 daily and tablet moxifloxacin 400 mg × 1 daily
Day 44	Completed oral antibiotics. Discharged home on Day 46
Day 54	Outpatient control 1 week after discharge—the patient is well and blood test results are normal
Afterwards	TTE 1 month after discharge—showed a normal LVEF of 50%, MVP *in situ*, no signs of IE
Follow-up	Colonoscopy showed benign changes in colon ascendens (tubular adenoma LG)

EEG, electroencephalogram; GP, general practitioner; ICU, intensive care unit; IE, infective endocarditis; i.v., intravenous; LVEF, left ventricular ejection fraction; MRI, magnetic resonance image; MVP, biological mitral valve prosthesis; PET/CT, positron emission tomography/computed tomography; TEE, transoesophageal echocardiography; TTE, transthoracic echocardiography.

Laboratory blood results (at admission) showed a haemoglobin level of 10.3 g/dL (normal range, 11.8–15.3 g/dL), white blood cells of 12.2 × 10^9^/L (normal range, 3.5–8.8 × 10^9^/L) with an overweight of neutrophilic predominance, a C-reactive protein (CRP) of 30 mg/L (normal range, <8 mg/L), a creatinine level of 110 µmol/L (normal range 45–90 µmol/L), and an estimated glomerular filtration rate (eGFR) of 38 mL/min 1.73 m^2^ (normal range >60 mL/min 1.73 m^2^).

Blood cultures (over 2 days, a total of 12 samples were drawn) showed a growth of *Streptococcus salivarius* subspecies in all 12 samples.

**Figure 1:**
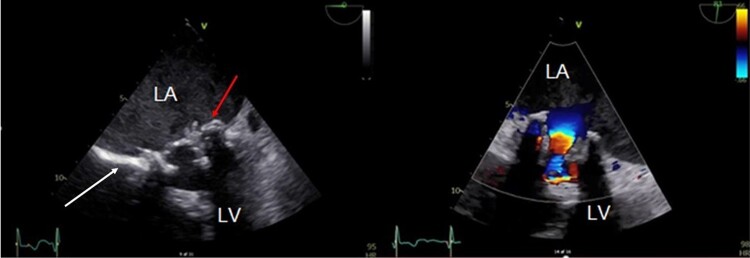
Transoesophageal echocardiography of Patient 1. The top arrow points at the mitral valve prosthesis and the vegetation. The lower arrow indicates the atrial septum. LV, left ventricle; RV, right ventricle.

Transoesophageal echocardiography showed a tricuspid aortic valve with no stenosis or regurgitation and the MVP *in situ* with no regurgitation. Thickened leaflets and fluttering vegetations were observed on both leaflets, mainly in the anterolateral part (*Figure 1*). A mean gradient of 11 mmHg was observed for mitral inflow through the prosthesis. The left ventricle was structurally normal with a normal LVEF. The left atrium was enlarged and dilated.

**Figure 2:**
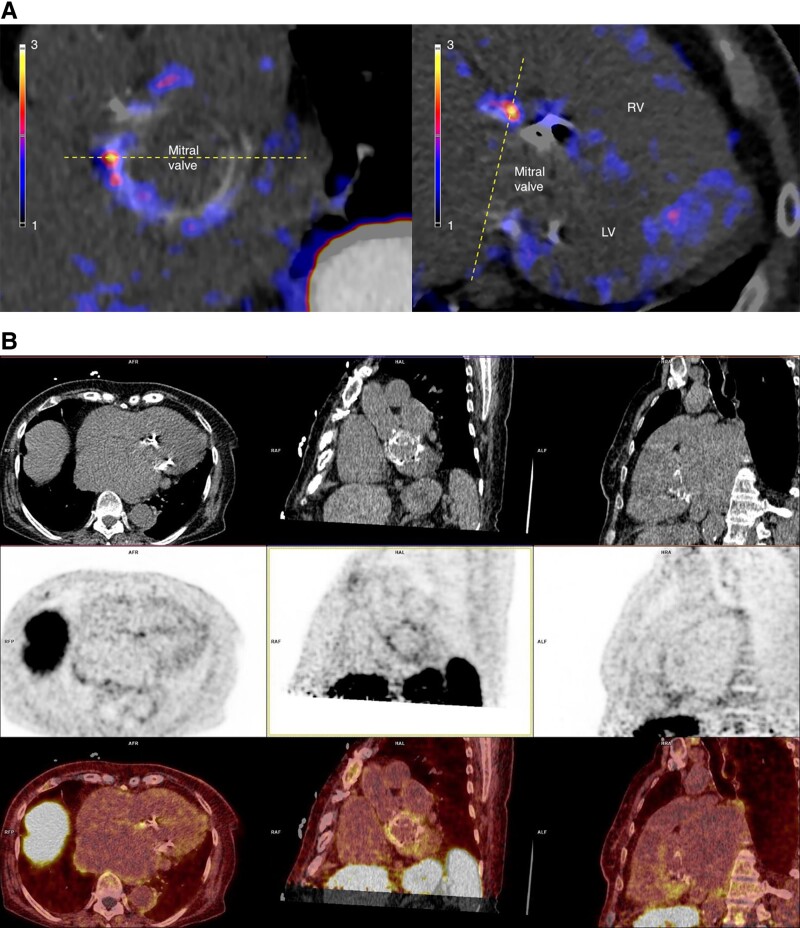
(*A*) [^64^Cu]Cu-DOTATATE PET/CT of Patient 1. Perpendicular positron emission tomography/computed tomography fusion images of the mitral valve revealing a focal high-tracer uptake of [^64^Cu]Cu-DOTATATE on the anterior aspect of the mitral valve prosthesis. The myocardium in the ventricle has very low tracer uptake. The dotted lines indicate two image planes. LV, left ventricle; RV, right ventricle. (*B*) Positron emission tomography/computed tomography images from Patient 1 showing an uptake of [^64^Cu]Cu-DOTATATE in cross-sectional, coronal, and sagittal views, respectively. Top row: computed tomographyCT, middle row: positron emission tomography images; bottom row: fused positron emission tomography /computed tomography images.

The patient underwent [^64^Cu]Cu-DOTATATE PET/CT, which revealed a focal high-tracer uptake on the anterior aspect of the MVP, especially around the commissures (*Figure 2A* and *B*). The background uptake of the tracer in the myocardium and the blood was low. No focal increased uptake was observed in the other valves.

It was not possible to perform a comparative [^18^F]FDG PET/CT, as the surgical decision was made after [^64^Cu]Cu-DOTATATE PET/CT, and the patient underwent surgery within 72 h due to large vegetations. A new biological MVP was inserted (Abbott Epic 31). The removed infected prosthesis showed no bacterial growth. The post-operative period was uncomplicated, and the patient was discharged home after 45 days in hospital and after completing the antibiotic treatment (in total, 42 days of intravenous therapy). The follow-up was similarly uncomplicated, and the patient returned to her habitual activities (such as food preparation, family visits, and bicycling) in a couple of months.

### Timeline: Patient 2

A 72-year-old male patient with a medical history of hypertension, hyperlipidaemia, ischaemic heart disease (acute myocardial infarction in 2017), mild mitral valve stenosis and moderate aortic valve stenosis, and gastrointestinal stromal tumour and who underwent Whipple surgery in 2017 was admitted to the emergency department for back pain, unspecific stomach pain, and subfebrile episodes. The most recent TEE (4 months before admission) showed a normal LVEF, a moderate aortic stenosis, and a calcified mitral valve. The physical examination revealed abdominal tenderness, but otherwise normal findings. The patient was married and self-reliant in daily activities. A detailed timeline of the patient’s progress during hospitalization is given in *Table 2*.

**Table 2:** Timeline Patient 2

Time	Event
Initial examination	The patient felt unwell with subfebrile episodes, with a weight loss of 4 kg in over ∼2 months before admission
Day 1	Admitted to the emergency department due to acute severe back pain for 4 days and fever. The initial suspected diagnoses were spondylodiscitis/endocarditis/abscess
Day 2	Blood cultures positive for *Enterococcus faecalis* in six out of eight blood culture bottles
Day 2	Bedside TTE: without visible vegetations, LVEF 50%. The patient was referred for a TEE
Day 4	TEE: native aortic valve with multiple large vegetations on all three cusps Antibiotic regimen, ampicillin, and linezolid
Day 4	MRI of the spine: inflammatory reaction at L5/S1 level. Suspected for spondylodiscitis
Day 4	Transferred to the tertiary centre for surgical assessment
Day 5	[^64^Cu]Cu-DOTATATE PET/CT was performed. Endocarditis team conference—referred for surgical aortic valve replacement
Day 7	Surgery with a biological aortic valve (23 mm Avalus biological aortic heart valve from Medtronic)
Day 7	Transferred to the ICU. Progress—no complications
Day 8	Extubated. Transferred to the cardiology ward
Day 10	Post-operative TTE showed a biological valve *in situ*, well positioned. LVEF 45%. Small pericardial effusion without haemodynamic effect
Day 11	The removed native heart valve showed a growth of *E. faecalis*
Day 11	[^18^F]FDG PET/CT—showed spondylodiscitis foci in the columna—confirmed MRI results
Day 12	Dental examination showed infectious foci at two site areas, which were extracted
Day 13	Transferred back to the local hospital for further treatment
Afterwards	The post-operative period was characterized by *Candida albicans* sternitis, which was treated with surgical drainage and anti-fungal medicine. The patient was also infected with COVID-19. After 80 days, the patient was stabilized and discharged home. However, the patient was readmitted 4 days later to the department of infectious diseases with sepsis and reoccurrence of *C. albicans*
Follow-up:	The patient died during admission (12 days after) of respiratory failure after a complication of sepsis

GP, general practitioner; MRI, magnetic resonance image; PET/CT, positron emission tomography/computed tomography; TTE, transthoracic echocardiography; TEE, transoesophageal echocardiography; LVEF, left ventricular ejection fraction.

Laboratory blood results (at admission) showed a haemoglobin level of 8.4 g/dL (normal range, 13.4–16.9 g/dL), white blood cells 14.4 × 10^9^/L (normal range, 3.5–8.8 × 10^9^/L) with an overweight of neutrophilic predominance, CRP 214 mg/L (normal range, <10 mg/L), creatinine 62 µmol/L (normal range, 60–105 µmol/L), and eGFR >90 mL/min 1.73 m^2^ (normal range, >60 mL/min 1.73 m^2^).

Blood cultures (over 2 days, a total of 8 samples were drawn) showed a growth of *Enterococcus faecalis* in six out of eight blood culture samples.

**Figure 3:**
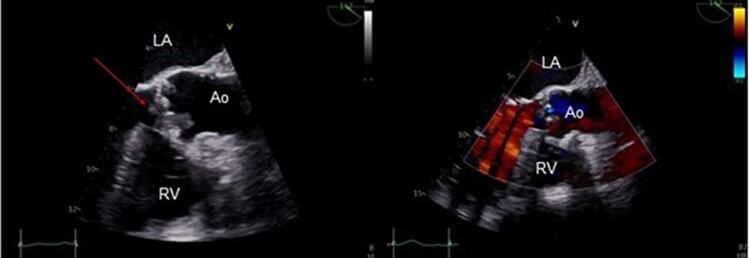
Transoesophageal echocardiography of Patient 2. The arrow indicates the aortic valve and the vegetations. Ao, aorta; LV, left ventricle; RV, right ventricle.

Transoesophageal echocardiography showed a tricuspid aortic valve with mild calcifications and large mobile vegetations on all three cusps—the largest being 15 mm × 0.5 mm (*Figure 3*) with moderate aortic regurgitation: The mitral valve was calcified with mild regurgitation. The left ventricle was structurally normal, with a mild reduction in the pump function (LVEF 50%).

**Figure 4:**
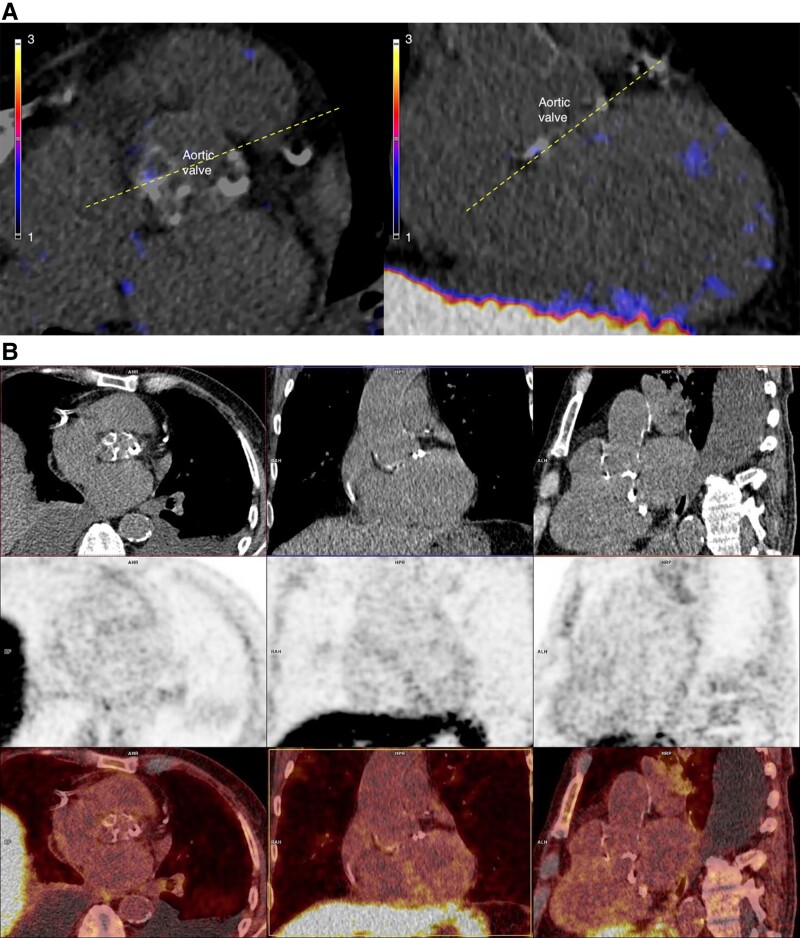
(*A*) [^64^Cu]Cu-DOTATATE positron emission tomography/computed tomography of Patient 2. Perpendicular positron emission tomography/computed tomography fusion images of the aortic valve without an increased uptake of [^64^Cu]Cu-DOTATATE. The myocardium in the ventricle has very low tracer uptake. The dotted lines indicate two image planes. LV, left ventricle; RV, right ventricle. (*B*) Positron emission tomography/computed tomography images from Patient 2 showing an uptake of [^64^Cu]Cu-DOTATATE in cross-sectional, coronal, and sagittal views, respectively. Top row: computed tomography images; middle row: positron emission tomography images; bottom row: fused positron emission tomography/computed tomography images.

The patient underwent [^64^Cu]Cu-DOTATATE PET/CT, which showed no significant uptake of the tracer at the site of infection in the native aortic valve (*Figure 4A* and *B*). The other three native valves were also without any focal tracer uptake. The background uptake in the blood pool was low, and the uptake in the normal myocardium was faint just above the blood level.

It was not possible to perform a comparative [^18^F]FDG PET/CT, as the surgical decision was made after [^64^Cu]Cu-DOTATATE PET/CT, and the patient underwent surgery within 48 h due to large vegetations. A biological aortic valve was inserted (23 mm Avalus biological aortic heart valve). The removed valve showed a growth of *E. faecalis*. The post-operative period was complicated and characterized by *Candida albicans* sternitis, which was treated with surgical drainage and anti-fungal medicine. Further, the patient had reoccurring episodes of delirium, anaemia, and thrombocytopaenia, which were treated accordingly. The patient was also infected with COVID-19. After 80 days (of which 50 days were devoted for intravenous antibiotic therapy for IE, and the remaining 30 days were devoted for antibiotic therapy for sternitis), the patient was stabilized and discharged home; however, he was readmitted 4 days later with sepsis and a reoccurrence of *C. albicans*. After 12 days of hospitalization, the patient died of respiratory failure as a complication of sepsis.

## Discussion

Correct and early detection of inflammatory foci is essential for effective treatment of patients with IE. Both presented cases fulfilled the modified Duke criteria and modified ESC criteria 2023 for definite IE.^5^ This case series represents the first description of [^64^Cu]Cu-DOTATATE PET/CT in two patients with IE.

Uptake was found in Patient 1 (PVE case), whereas Patient 2 (NVE case) showed no uptake. Histologically, it has previously been described that patients with prosthetic valves have a higher presence of macrophages on the valve compared with NVE, where neutrophil granulocytes are more predominant.^13,14^ This could explain why Patient 1 showed tracer uptake in [^64^Cu]Cu-DOTATATE PET/CT, whereas Patient 2 showed no uptake.

Prosthetic valve endocarditis is an uncommon but severe complication of surgical and transcatheter valve replacement with high mortality rates.^2,15,16^ The diagnosis can be challenging, and current guidelines recommend TTE, TEE, cardiac CT, and [^18^F]FDG PET/CT for diagnosing PVE.^5^ For the two described cases, TEE was diagnostic and the diagnosis of definite IE was clear. However, the [^64^Cu]Cu-DOTATATE PET/CT scan was performed as part of a protocolized proof-of-concept study for research purposes. Neither patient underwent a comparable [^18^F]FDG PET/CT due to clinical circumstances (large vegetations) and due to the fact that the surgical decision was made by the Endocarditis Team shortly after the [^64^Cu]Cu-DOTATATE PET/CT scan.

Compared with the [^18^F]FDG tracer, [^64^Cu]Cu-DOTATATE has a clear advantage as it has no significant physiological uptake in the normal myocardium and thus does not require any dietary restrictions or fasting before the scan. Positron emission tomography/CT with [^18^F]FDG has significant limitations regarding specificity and reproducibility as it is a glucose analogue taken up by metabolically active cells and is not specific for inflammatory cells.^12^ [^18^F]FDG PET/CT has a sensitivity of 84% for PVE, and the specificity is 71%.^6^ Studies have indicated that [^64^Cu]Cu-DOTATATE is superior to [^18^F]FDG for the detection of inflammation in atherosclerotic plaques and identification of culprit lesions, where pro-inflammatory macrophages also are predominant.^11^ Thus, our hypothesis is that ^64^[Cu]Cu-DOTATATE PET/CT can potentially have a higher specificity for PVE and an increased negative predictive value for PVE.

The case series has limitations. Firstly, it was restricted to two patients, thus limiting the clinical implication. Secondly, the anatomical difference and the localization of IE differed between the two patient cases.

In conclusion, this is the first case series describing the application of the novel tracer [^64^Cu]Cu-DOTATATE in two patients with IE. [^64^Cu]Cu-DOTATATE PET/CT showed a positive uptake in PVE, but not in NVE. Compared with [^18^F]FDG PET/CT, [^64^Cu]Cu-DOTATATE PET/CT has notable advantages such as no myocardial uptake and no preparatory need ahead of the scan. In addition, [^64^Cu]Cu-DOTATATE PET/CT is specific for pro-inflammatory macrophages. Therefore, the hypothesis is that [^64^Cu]Cu-DOTATATE PET/CT has the potential to be a better diagnostic tool for the identification of PVE. However, prospective studies are warranted to validate [^64^Cu]Cu-DOTATATE PET/CT and its safety in patients with IE, preferably studies with systematic head-to-head comparisons between [^64^Cu]Cu-DOTATATE PET/CT and [^18^F]FDG PET/CT in patients with IE.

## Supplementary Material

ytae431_Supplementary_Data

## Data Availability

The data underlying this article are available in the article and in its online Supplementary material.

## Lead author biography

Dr Katra Hadji-Turdeghal, MD, graduated as a medical doctor from the University of Copenhagen. She completed an internship in cardiology and is currently a PhD fellow at the Department of Cardiology, Copenhagen University Hospital, Rigshospitalet, Denmark.

## Notes

**Consent:** Both patients provided written informed consent for the data in this manuscript to be published in line with COPE guidelines.

**Funding:** This work was supported by the Danish Heart Foundation.
